# Uncommon form of normal-tension glaucoma

**Published:** 2017

**Authors:** Mihail Zemba, Tatiana Danilova, Liliana Pulbere, Alina-Cristina Stamate

**Affiliations:** * Department of Ophthalmology, “Dr. Carol Davila” Central University Military Emergency Hospital, Bucharest, Romania; ** “Carol Davila” University of Medicine and Pharmacy, Bucharest, Romania; *** Arena Med Clinic, Bucharest, Romania

**Keywords:** traumatic brain injury, induced coma, normal-tension glaucoma

## Abstract

****Aim**:**

To present diagnostic particularities, assessment of prognosis, and the need for treatment in a case of normal-tension glaucoma.

****Methods**:**

- presentation of clinical changes and investigations supporting the diagnosis; - careful anamnesis that disclosed new elements, useful for the evaluation of the case.

****Results**:**

after a two-year follow-up period, we can ascertain that the optic atrophy is non-progressive.

****Conclusions**:**

the assessment of risk factors and a rigorous anamnesis were significant for the establishment of prognosis and need for treatment.

## Introduction

Since its description by von Graephe in 1857 [**1**], normal-tension glaucoma has remained a diagnostic and therapeutic challenge for ophthalmologists.

It is a clinical condition associated with excavated optic disc and visual field defects, both compatible with glaucoma, although the intraocular pressure is statistically normal. The clinical course of the disease is quite similar to primary open-angle glaucoma.

The pathogenesis of normal-tension glaucoma is still unclear. Progressive optic neuropathy with a statistical normal intraocular pressure indicates a vascular insufficiency. This mechanism is supported by the association with migraine, peripheral vasospasm and recurrent optic disc hemorrhages. On the other hand, the association with peripapillary atrophy and myopia suggests a deficiency at the level of short posterior ciliary circulation [**2**].

## Case report

We describe the case of a 33-year-old male patient who first presented to our clinic in February 2015 with decreased visual acuity in the left eye.

Medical history showed a traumatic brain injury due to a war explosion in January 2012. He suffered a severe craniocerebral trauma with fracture of the temporal bone and an epidural hematoma, which was drained three days after admission.

Two months later, at discharge, the **diagnosis** was:

**1. Traumatic brain injury due to explosion**

**2. Mixed hypoacusis in the left ear (conductive and sensorineural)**

**3. Posttraumatic left peripheral facial palsy. Lagophthalmos**

From 2012 to 2015, he underwent different kinds of surgeries:

- **neurosurgical:** drainage of hematoma,

- **ENT:** 8 surgeries between 2012 and 2014 for ear drum reconstruction, myringotomy and removal of bone fragments that narrowed the ear meatus,

- **ophthalmological:** total blepharorrhaphy, partial opening of the blepharorrhaphy in March 2013 and total opening of the blepharorrhaphy in December 2014.

**Ophthalmologic examination revealed:**

**OD:**

**Fig. 1:**
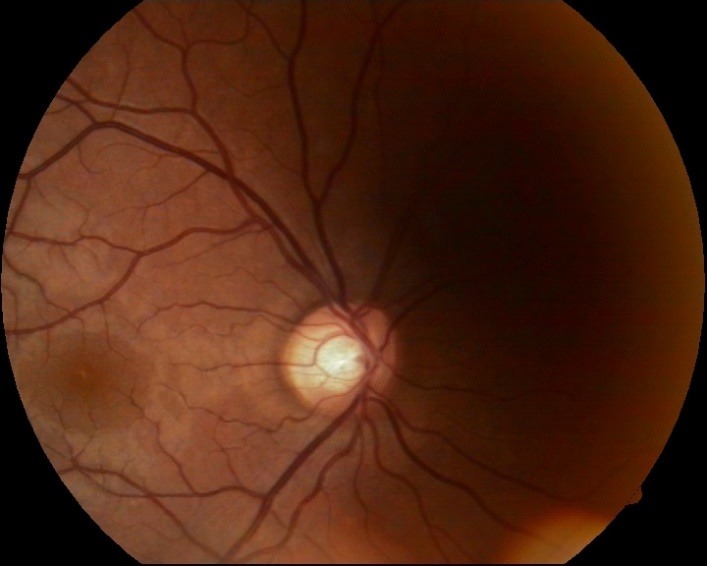
Fundus image OD

- visual acuity: 1 without correction

- refraction: +0.25/- 0.5* 11˚

- intraocular pressure: 13 mmHg NCT

- slit lamp examination: normal anterior segment

- fundus examination: well defined contour and normal color of the optic disc, C/ D ratio of 0.4, neuroretinal rim respecting the ISNT rule, normal macula and retinal vessels (**Fig. 1**).

**OS:**

**Fig. 2:**
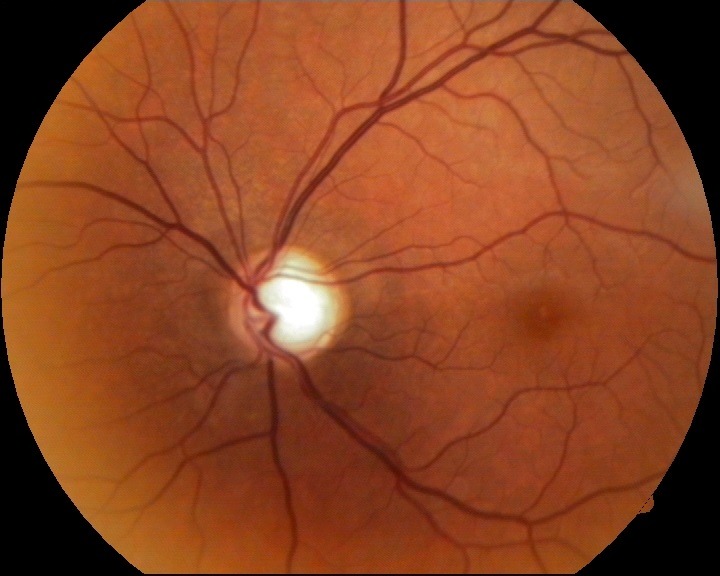
Fundus image OS

- visual acuity: 0.8 without correction

- refraction: +0.00/ -0.25*17˚

- intraocular pressure: 12 mmHg NCT

- slit lamp examination: discrete inferior, superficial corneal opacities

- fundus examination: well defined contour and pale color of the optic disc, C/ D ratio of 0.9, superior and temporal narrowing of the neuroretinal rim and absent inferiorly, normal macula and retinal vessels (**Fig. 2**).

**Diagnosis at this stage of the evaluation:**

OD: Normal

OS: Optic disc atrophy. Normal-tension glaucoma - under observation

**Fig. 3:**
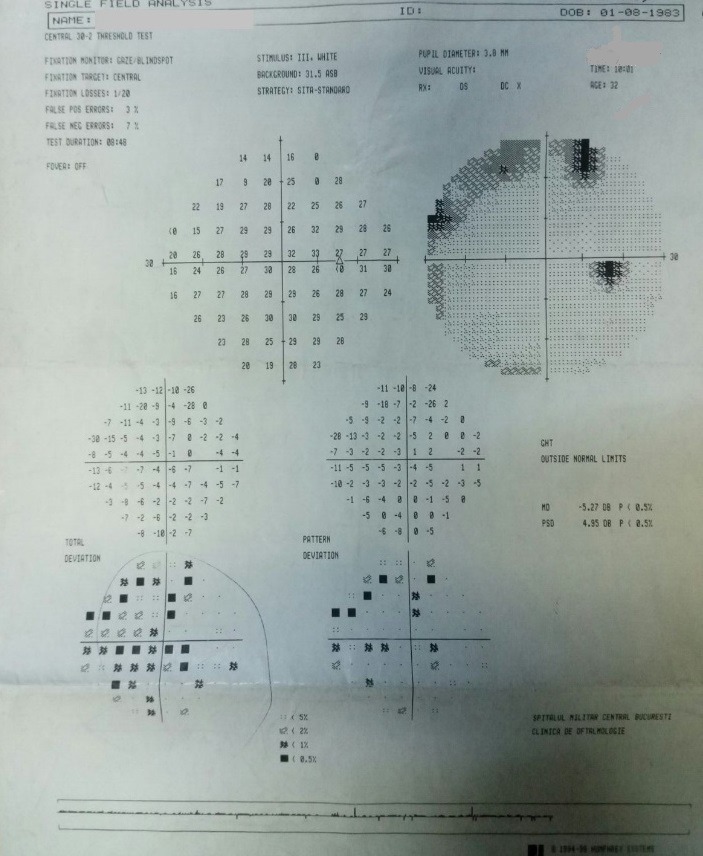
Visual field OD

**Fig. 4:**
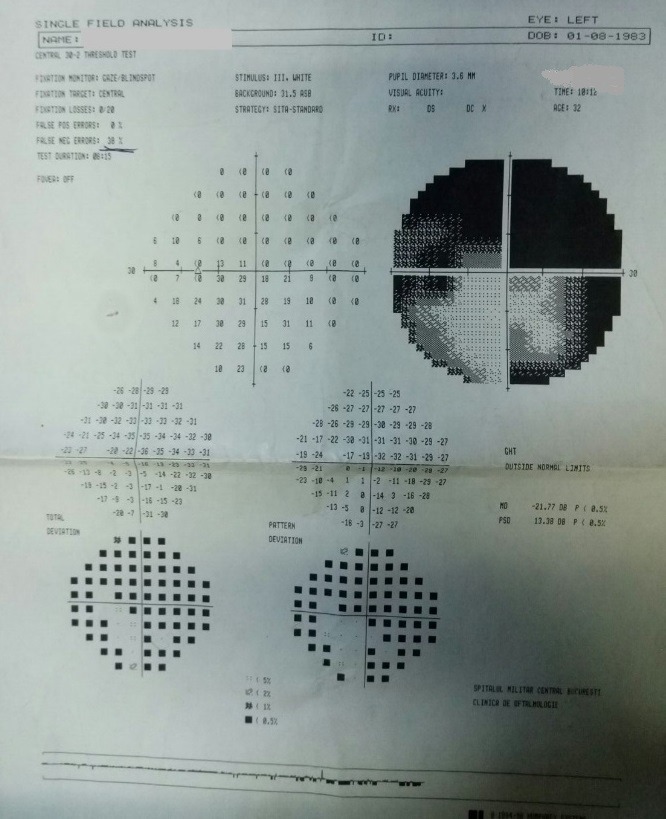
Visual field OS

**Fig. 5:**
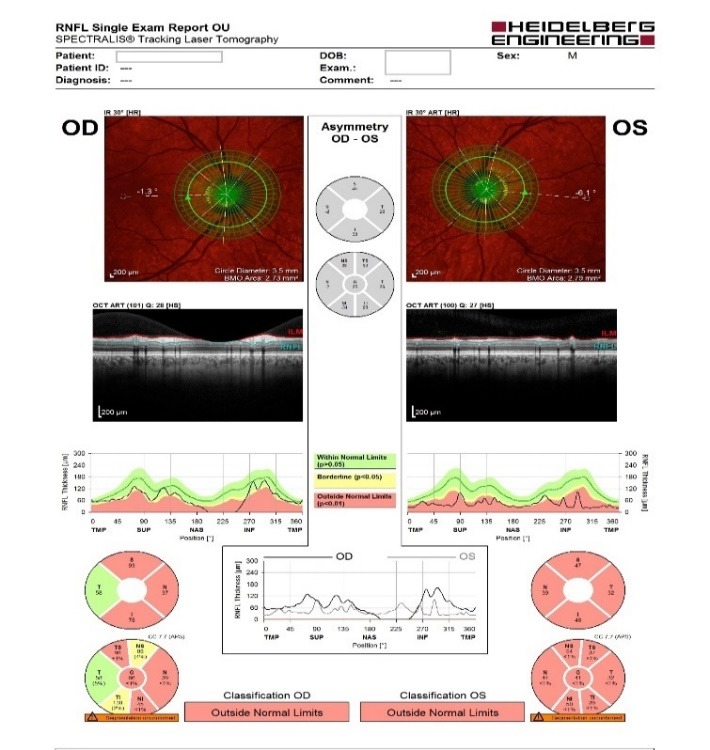
RNFL analysis

**Fig. 6:**
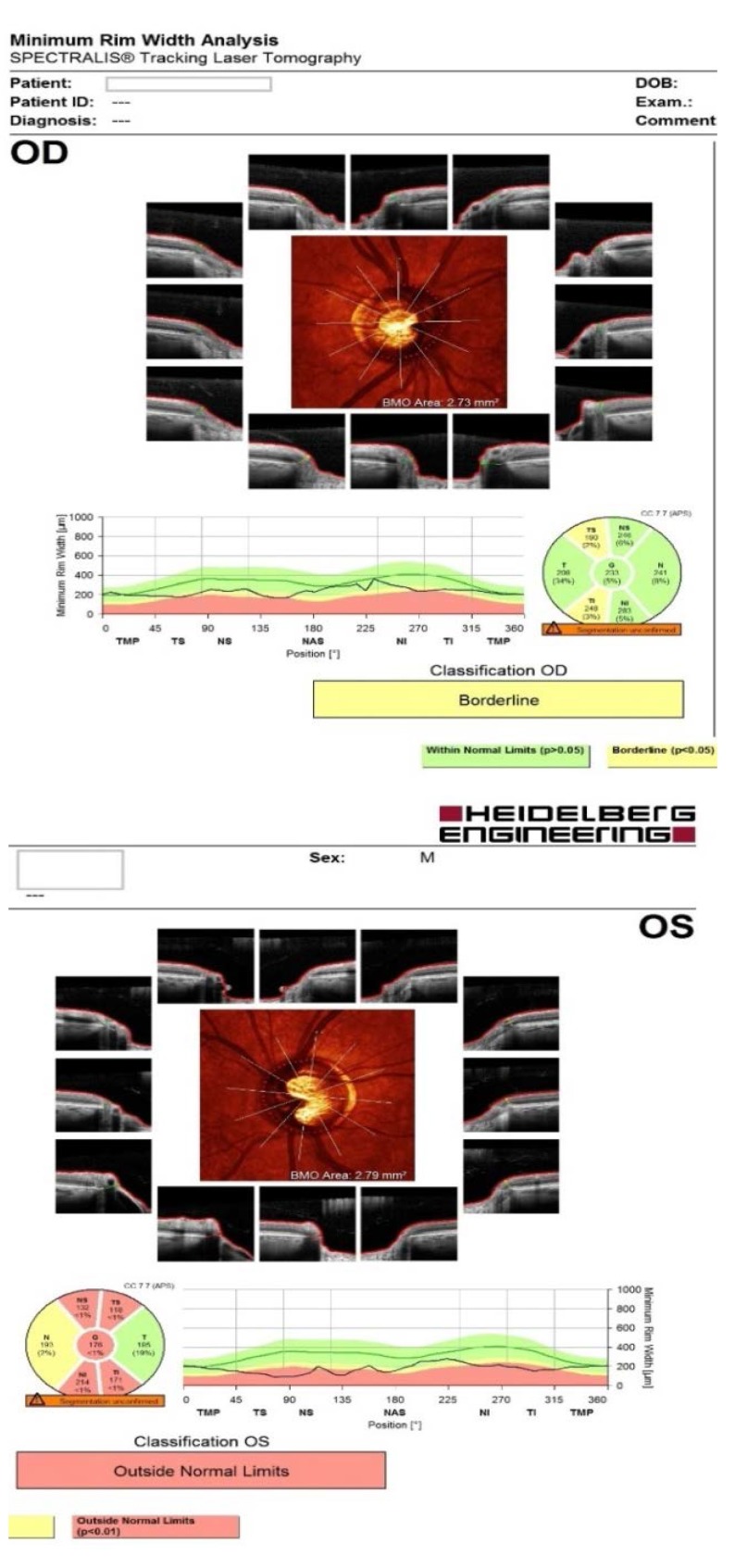
RNFL analysis at the level of Bruch membrane opening

**Investigations**:

- **pachymetry:** OD: 540 microns, OS: 541 microns

- **visual field examination **(Humphrey Field Analyzer, Zeiss): OD: small peripheral defects in all four quadrants; MD: -5.27 dB; but with low reliability indices (**Fig. 3**), OS: severe decrease in retinal sensitivity, MD: -21.7 dB; glaucoma hemifield test: outside normal limits; subtle inferior arcuate defect, deep superior arcuate defect, no altitudinal defect (**Fig. 4**).

- **retinal nerve fiber layer (RNFL) analysis:** OD: decreased thickness in three quadrants, OS: decreased thickness in all four quadrants (**Fig. 5**).

- **retinal nerve fiber layer (RNFL) analysis at the level of Bruch membrane opening:** OD: mild temporal, superior and inferior thinning of the RNFL (borderline changes), OS: important thinning of the RNFL (outside normal limits) (**Fig. 6**).

**Diagnosis: OU: Normal-tension glaucoma**

Our diagnosis was supported by the following:

- intraocular pressure always below 15 mmHg,

- structural changes: 

• OS: specific glaucomatous optic disc cupping,

• OU: changes in retinal nerve fiber layer thickness, 

- functional changes: characteristic glaucomatous changes in OS, borderline changes in OD; considering Hodapp’s classification, glaucoma changes in OS were advanced (MD > -12 dB; > 37 points with p< 5%; > 22 points with p< 1%; < 15 dB sensitivity in the 5 central degrees in both hemifields).

**Differential diagnosis:**

- primary open-angle glaucoma, but intraocular pressure was always under 15 mmHg;

- anterior ischemic optic neuropathy with optic disc atrophy, but the visual field defect was not specific for this disease.

**Treatment:**

Our decision was to treat the patient and our approach consisted in combining prostaglandin analogues (i.e. latanoprost, once daily at bedtime) and neuroprotective agents (administered twice daily).

## Discussions

The first discussion concerned the reasons why the treatment was started. Collaborative Normal Tension Glaucoma Study concluded that the level of pressure influenced the course of normal-tension glaucoma, as evidenced by a slower rate of incident visual field loss in cases with 30% or more lowering of IOP: 

- at 3 years:

• 20% of those treated had progression 

• 40% of those untreated had progression 

- at 5 years:

• 20% of those treated had progression 

• 60% of those untreated had progression [**3**].

We tried to analyze all the risk factors for normal-tension glaucoma and most of them were not present in our patient:

- age: he was under 35 years

- race: he was Caucasian

- myopia: no refractive error

- family history of glaucoma: negative

- spikes of high intraocular pressure: negative

- cardiovascular risk factors: systemic hypertension, systemic hypotension, nocturnal variations of blood pressure: negative

- vasospasm: migraine, Raynaud syndrome: negative

- sleep apnea syndrome: negative

- sensorineural hypoacusis: positive, but the cause was traumatic, not vascular

- silent cerebral infarct.

However, the turning point in our evaluation was that our anamnesis revealed that the patient had been in an induced coma for 6 weeks after his traumatic brain injury in 2012.

The induced coma is a therapeutic procedure that tries to protect the brain after a severe trauma. The drugs used are barbiturates, such as pentobarbital or thiopental [**4**]. They reduce the metabolic rate of the brain tissue and the cerebral blood flow. This way, the blood vessel in the brain narrowed and the amount of space occupied by the brain was reduced, so the intracranial pressure decreased. The aim was that by relieving the swelling and decreasing the intracranial pressure, some brain damages could be avoided [**5**,**6**].

The main risk of induced coma is severe blood hypotension. This can induce a very important decrease in optic blood flow. So, in some way, we can say that our patient had an “acute form of normal-tension glaucoma”, due to a significant and relatively long (weeks) drop in optic nerve blood flow, that resulted in optic disc atrophy.

**Further issues to be discussed:**

**1. Why did the patient seek medical advice so late, in 2015?**

The answer was the blepharorrhaphy. It was complete between January 2012 and March 2013 and partial, only temporally, between March 2013 and December 2014. This hindered a proper fundus examination of the left eye and also the patient had a quite good visual acuity and the visual field defects were obstructed by the blepharorrhaphy.

**2. Was the treatment necessary?**

The patient had no risk factor for normal-tension glaucoma and the main cause of his problem – the induced coma – was no longer a cause. Therefore, we thought that the need for treatment was doubtful. However, at the patient insistence, we decided to continue the treatment and the follow-up.

**Follow-up at three months:**

**Fig. 7:**
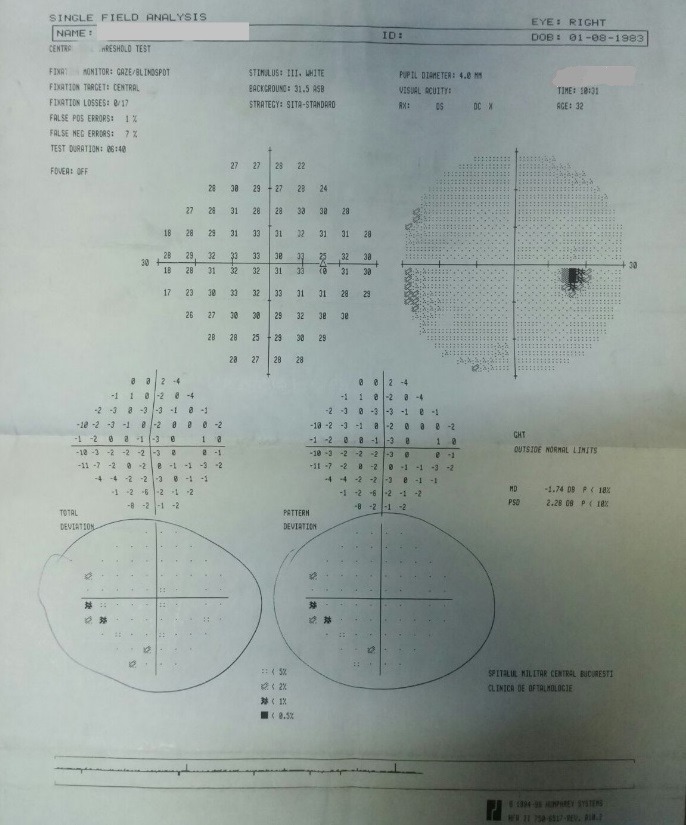
Visual field OD – follow-up at three months

**Fig. 8:**
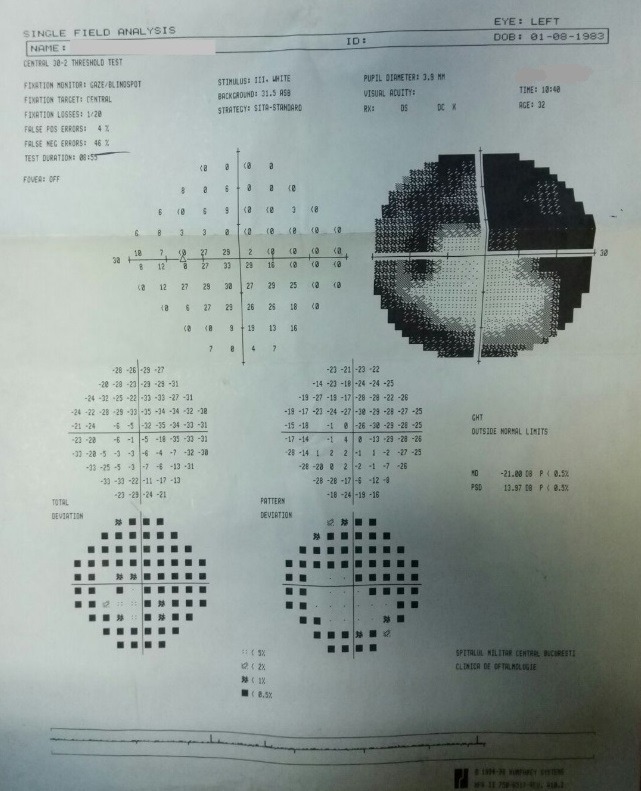
Visual field OS – follow-up at three months

- intraocular pressure: OD: 11 mmHg (< 20% drop in intraocular pressure), OS: 11 mmHg (< 10% drop in intraocular pressure)

- visual field: OD: the aspect improved, due to a better cooperation (**Fig. 7**), OS: the aspect was almost the same (**Fig. 8**).

**Fig. 9:**
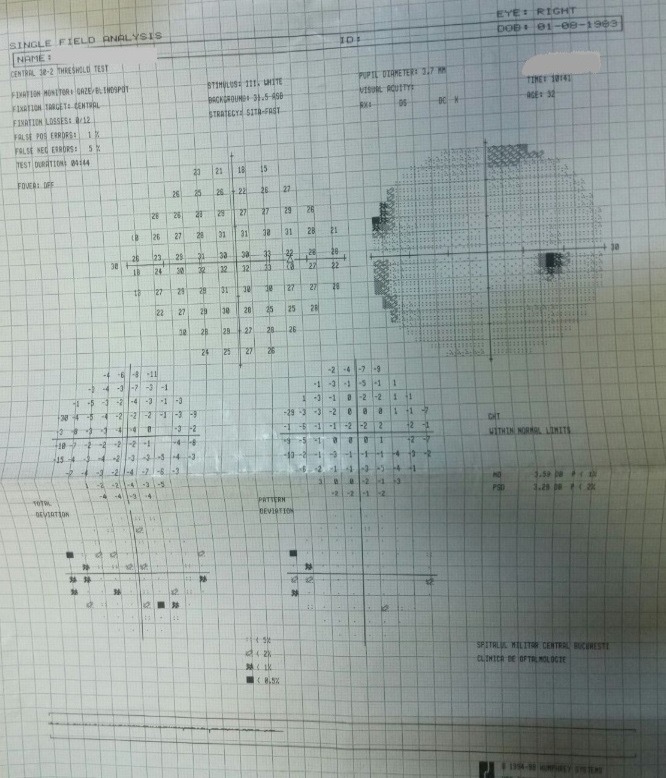
Visual field OD – follow-up at ten months

**Fig. 10:**
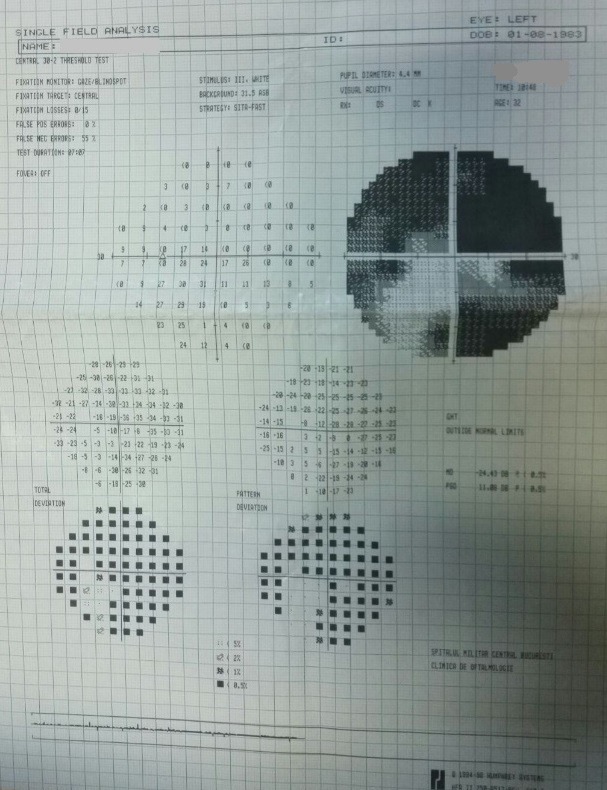
Visual field OS – follow-up at ten months

**Fig. 11:**
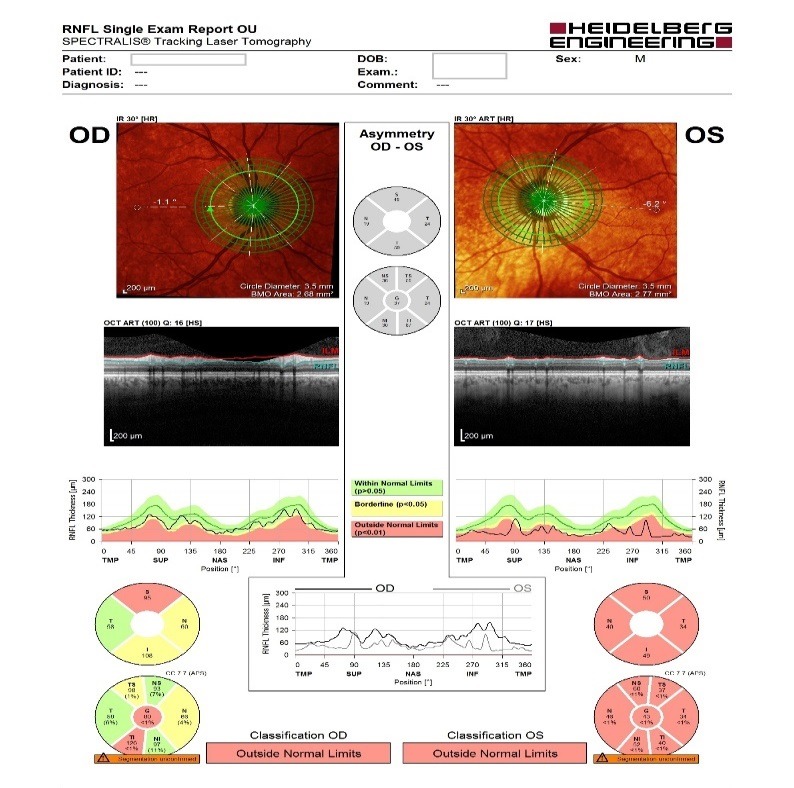
Retinal nerve fiber layer analysis – follow-up at ten months

Follow-up at ten months:

- intraocular pressure: OD: 12 mmHg (< 10% drop in intraocular pressure), OS: 10 mmHg (< 20% drop in intraocular pressure)

- visual field: OD: the aspect was constant (**Fig. 9**), OS: the aspect was almost the same (**Fig. 10**)

- retinal nerve fiber layer analysis showed no important changes: OD borderline, OS: outside normal limits, with similar RNFL thickness in all quadrants as ten months before (**Fig. 11**).

**Fig. 12:**
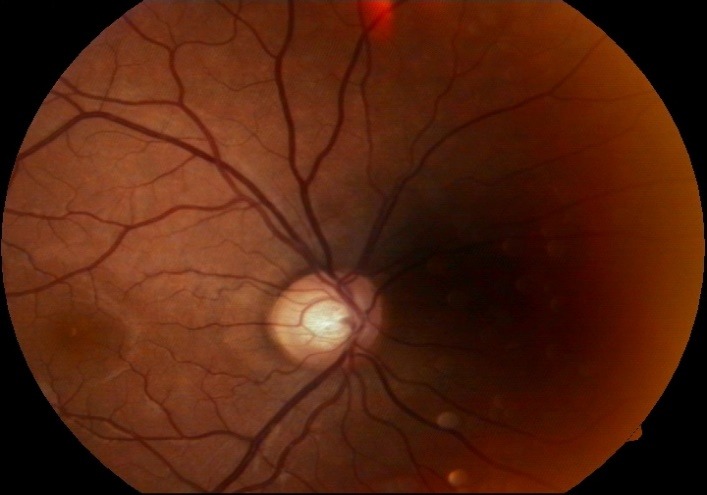
Fundus examination OD - follow-up at 16 months

**Fig. 13:**
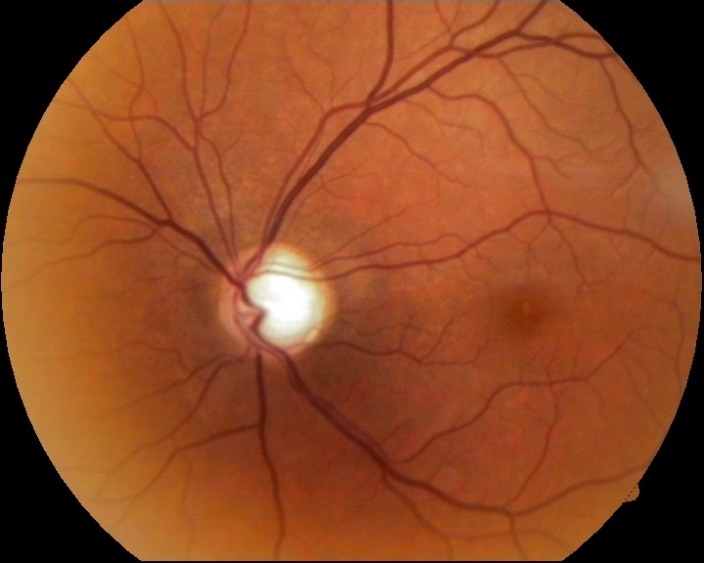
Fundus examination OS - follow-up at 16 months

**Fig. 14:**
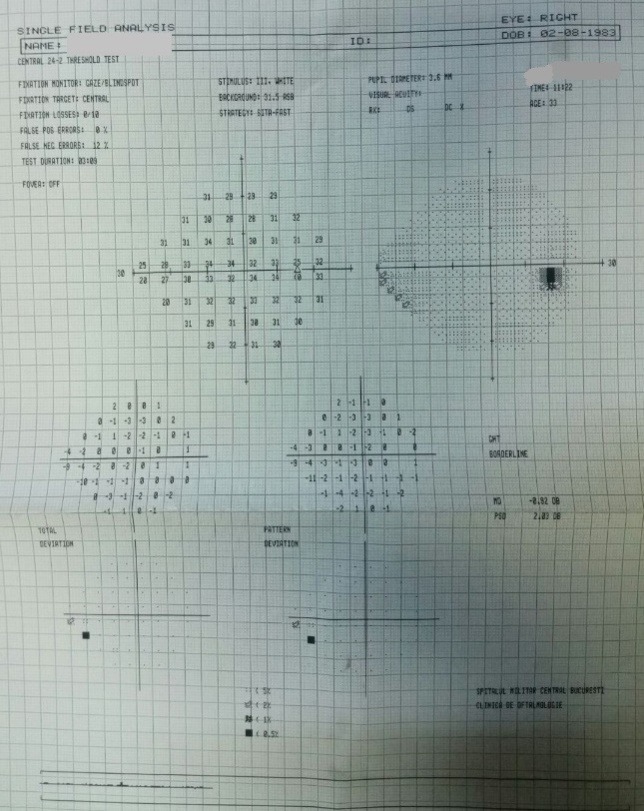
Visual field OD - follow-up at 16 months

**Fig. 15:**
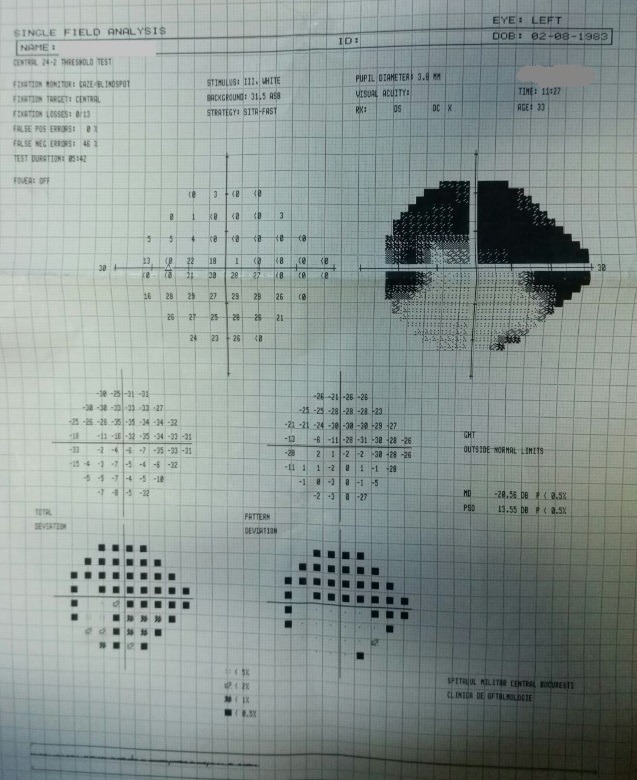
Visual field OS - follow-up at 16 months

**Fig. 16:**
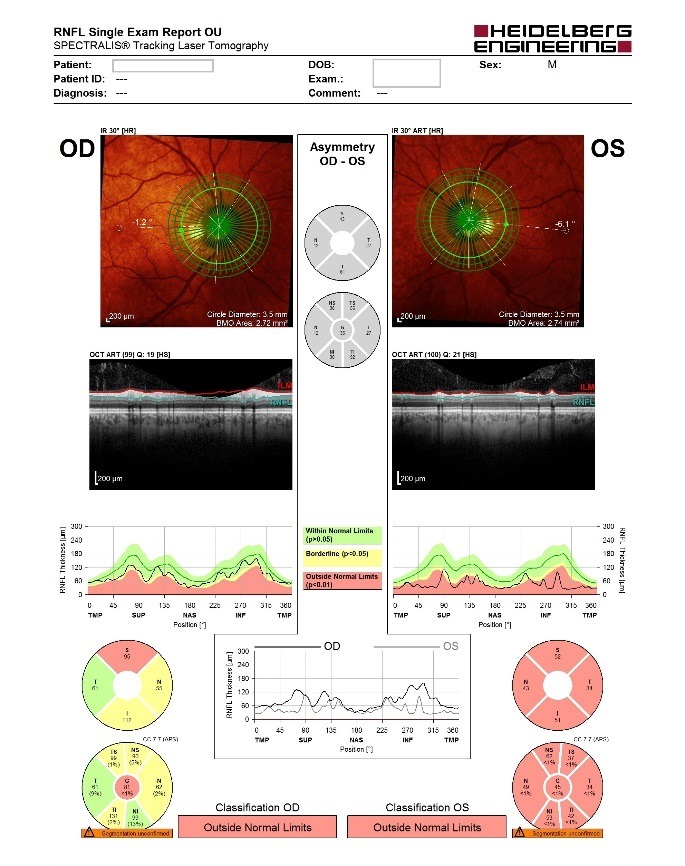
Retinal nerve fiber layer analysis - follow-up at 16 months

Follow-up at sixteen months:

- intraocular pressure: OD: 13 mmHg, OS: 12 mmHg 

- fundus examination: OU: no changes (**Fig. 12**, **Fig. 13**)

- visual field: OD: the aspect was constant (**Fig. 14**), OS: the aspect was almost the same (**Fig. 15**)

- retinal nerve fiber layer analysis showed no important changes: OD: borderline, OS: outside normal limits with similar RNFL thickness in all quadrants as 16 months before (**Fig. 16**).

Considering that the RNFL thickness remained similar and that the visual field defects did not deteriorate for the entire follow-up period (16 months), we decided to stop the antiglaucomatous treatment (eye drops) and leave the patient only on neuroprotective treatment.

**Follow-up at 23 months:**

**Fig. 17:**
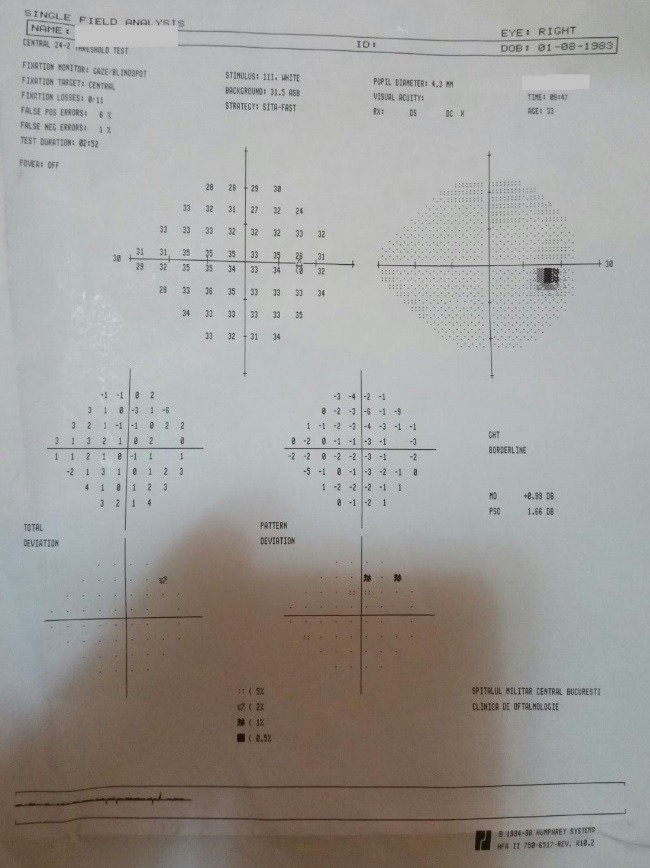
Visual field OD – follow-up at 23 months

**Fig. 18:**
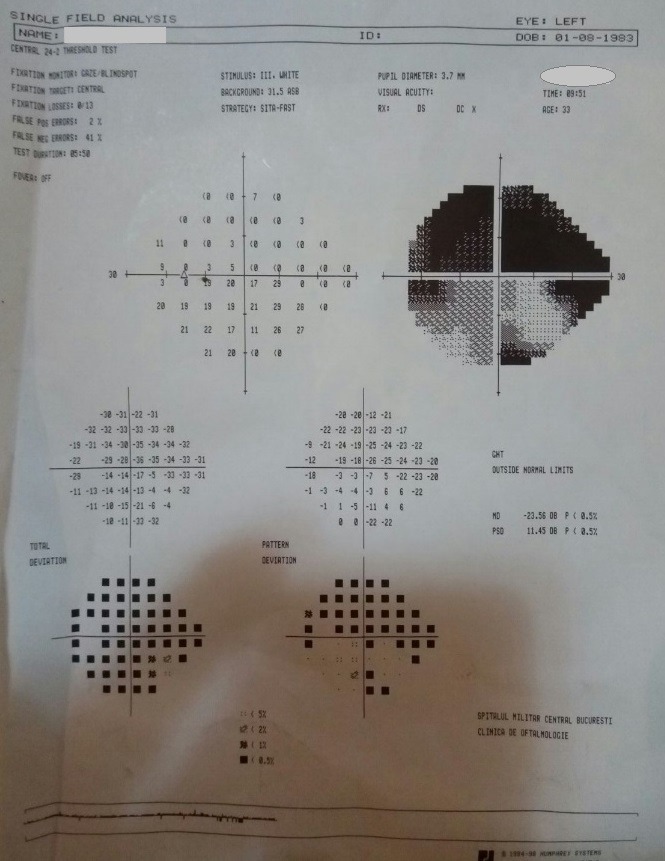
Visual field OS – follow-up at 23 months

**Fig. 19:**
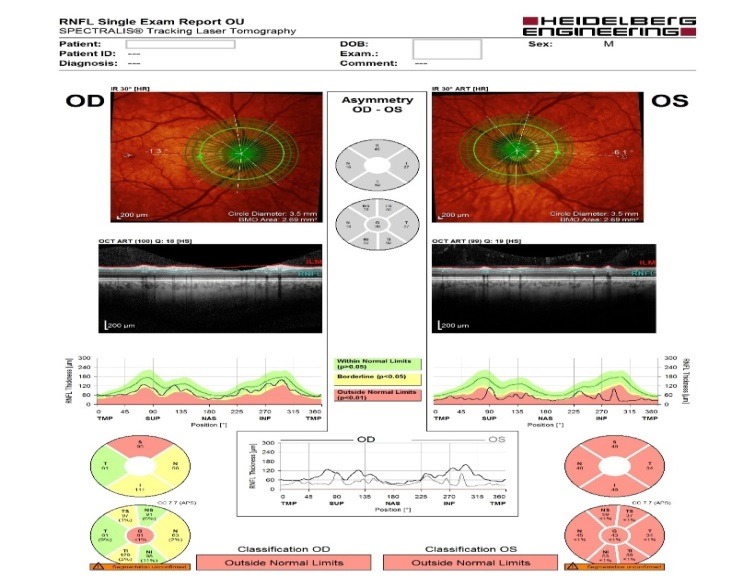
Retinal nerve fiber layer analysis – follow-up at 23 months

- visual acuity: OD: 1 without correction, OS: 0.8 without correction

- intraocular pressure: OD: 13 mmHg, OS: 12 mmHg

- fundus examination: C/ D ratio: OD: 0.4, OS: 0.9

- visual field: no significant changes (**Fig. 17**, **Fig. 18**)

- retinal nerve fiber layer analysis showed no important changes (**Fig. 19**).

After two years of follow-up, there were no changes in visual acuity, fundus examination, visual field, or retinal nerve fiber layer thickness. We consider that the patient’s prognosis is good and most probably he has a stationary impairment of visual function in OS, but not a progressive disease, as is normal-tension glaucoma. Maybe, in this case, the correct diagnosis should be: OU: Secondary optic nerve atrophy due to induced coma (OS>OD).

## Conclusions

1. Normal-tension glaucoma is a difficult diagnosis that requires multiple examinations.

2. After the evaluation of the risk factors, a careful anamnesis can offer information about the cause of the optic nerve atrophy.

3. The prognosis for this young patient changed for the better, because, after a rigorous follow-up and taking into account his medical history, his disease can be considered non-progressive. 

4. Quality of life improved for the patient, because he no longer requires chronic topical therapy and he no longer has to worry about the risk of going blind.

**Disclosures**

None of the authors has any financial or proprietary interests to disclose.
